# Case report: Minimal manifestations of mucous membrane pemphigoid in a young adult

**DOI:** 10.3389/fmed.2022.1052145

**Published:** 2022-11-17

**Authors:** Franziska Schauer, Federica Casetti, Dimitra Kiritsi

**Affiliations:** Department of Dermatology, Medical Center–University of Freiburg, Faculty of Medicine, University of Freiburg, Freiburg, Germany

**Keywords:** blistering disorder, skin fragility, urethral erosion, autoimmune disease, hemidesmosome, BP230

## Abstract

A male patient presented to our department at the age of 23 suffering from recurrent painful erosions in the urethral outlet area. In closer clinical examination gingival erosions, primarily around the teeth were identified as well. Indirect immunofluorescence on salt split skin with epidermal IgG deposition and positive anti-BP230 IgG ELISA diagnostics hinted toward the presence of mucous membrane pemphigoid (MMP). Direct immunofluorescence from oral mucosa confirmed the diagnosis. MMP in young adulthood is an underdiagnosed disease and latency of diagnosis was around 4 years in our case. Treatment with systemic glucocorticosteroids and dapsone led to clinical remission, prohibiting the development of MMP manifestations in further organs and complications associated with the disease, e.g., scar formation and miction problems.

## Introduction

Mucous membrane pemphigoid (MMP) is an autoantibody-mediated subepidermal blistering disease with predominant involvement of mucous membranes and tendency of scarring (1). The scarring can interfere with the patients’ normal functions, e.g., vision, food intake, miction, and sexual intercourse, thus have a serious impact for their quality of life (2). Larynx involvement might even be fatal due to breathing impairment (3). The diagnosis is based on clinical findings and the detection of autoantibodies against known autoantigens at the basement membrane, primarily BP180, BP230, laminin 332, and collagen VII (4). Direct and/or indirect immunofluorescence diagnostics, as well as ELISA for the specific proteins and immunoblotting are essential for correct diagnosis (4). We here present a male patient with only few erosions on his gingiva and urethra and minimal inflammatory signs of the disease, who was diagnosed having an MMP and responded to immunosuppressive treatment.

## Case report

**FIGURE 1:**
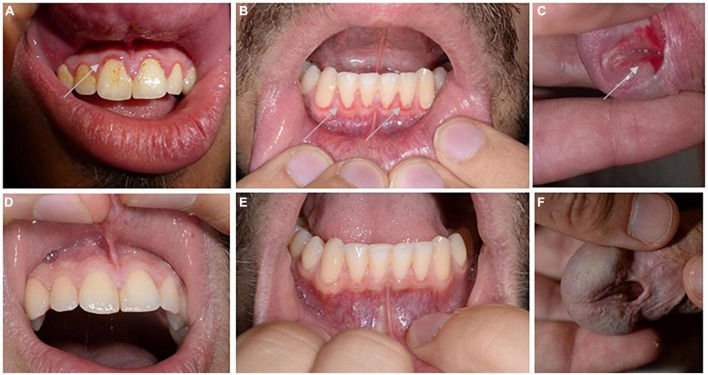
Clinical picture. Discrete swelling and erosions (highlighted by arrows) around the gingival fringes of the upper **(A)** and lower tooth space **(B)**, as well as a painful ulcer at the urethral entrance were identified at initial presentation **(C)**. Inconspicuous mucosal findings of the upper gingiva **(D)** and lower gingiva **(E)** and urethral outlet **(F)** followed initiation of therapy with prednisolone combined with dapsone.

A 23-year-old male patient from middle Europe suffered from recurrent, painful erosions in the urethral outlet area since around 4 years. Due to congenital hypospadias in the urethral area, he was regularly examined by urologists. The persistence of the mucosal lesions resulted in referral to our Dermatology department. Clinical examination showed an ulcer on the urethral outlet area, but also few erosions on the gingiva, which mimicked a mild gingivitis (Figure 1). He was not hampered by the gingival lesions in his everyday life. He was otherwise healthy, without any medication. Sexually transmitted disorders had been ruled out.

**FIGURE 2:**
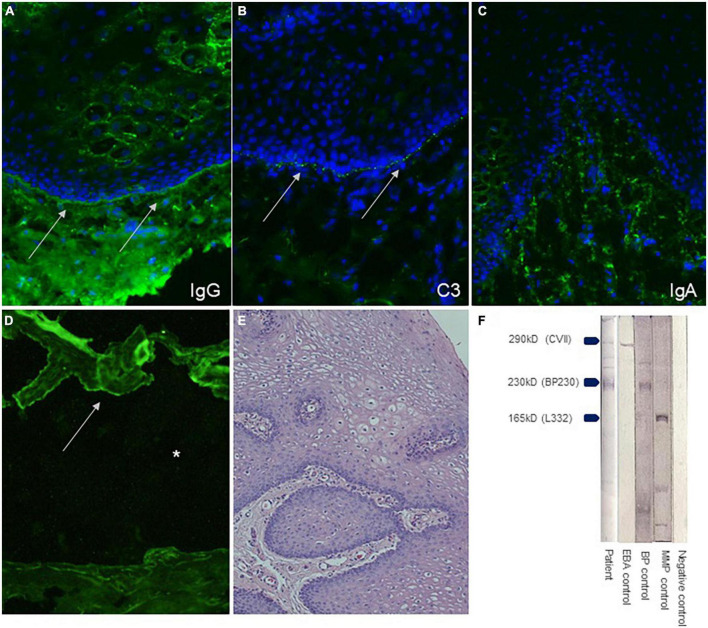
Direct immunfluorescence (DIF) of buccal mucosa shows linear IgG and C3 depositions at basement membrane zone, each indicated by arrows **(A,B)**. DIF found no reactivity for IgA **(C)**. Indirect immunofluorescence on 1M split human skin using patient’s serum shows epidermal staining of IgG **(D)**. Histopathology of buccal mucosa of the patient (hematoxylin-eosin stain, 200x magnification) **(E)**. Immunoblot analysis revealed IgG autoantibodies against BP230 and slightly also against collagen VII **(F)**.

We initiated specific diagnostics for autoimmune blistering disorders. The indirect immunofluorescence on salt split skin showed epidermal IgG reactivity at blister roof, compatible with an active pemphigoid disorder (Figure 2A). In addition, an isolated positive BP230 ELISA at 85 U/ml (cut of > 9 U/ml) was identified, while NC16A BP180 ELISA was negative (MBL, Nagoya, Japan). The subsequently performed analysis from a biopsy of unaffected buccal mucosa revealed IgG and C3 deposition at the dermal-epidermal junction in the direct immunofluorescence (Figures 2B–D). Immunoblotting analysis using human epidermal extract as substrate showed IgG immunoreactivity with BP230 and slightly also collagen VII (Figure 2F). IgA autoantibodies did not react in any analysis. Histological examination taken from buccal mucosa yielded a hyperplastic, non-keratinizing, mucosal epithelium without cleavage (Figure 2E). No inflammatory infiltrate was detectable in the dermis nor evidence for PAS-positive fungal elements were identified. The patient was treated with oral prednisolone 0.3 mg/kg body weight and oral dapsone 50 mg/day (0.8 mg/kg body weight). Lesions of the patients healed with topical tacrolimus 0.1% ointment for the urethral area and triamcinolone acetonide 0.1% cream for the gingiva after 8 weeks.

## Discussion

Mucous membrane pemphigoid (MMP) is a chronic and usually blistering disorder with about 2/million inhabitants/year being diagnosed in central Europe (5). In general, MMP can affect multiple mucosal sites, but oral mucosa is most frequently involved, followed by ocular, anogenital, nasopharyngeal, laryngeal, and esophageal sides (5, 6). It is considered a disease of the elderly with disease onset typically within the 6th decade of life (7). In young adulthood it is rarely described and sometimes associated with trauma (8). On the other hand, vulvar or urethral MMP has been described in children as well, while the genital area appears to be more commonly affected than other mucosal surfaces at young age (9). Altogether, less than 20 cases of isolated genital MMP have been reported in the literature, notably with either children or geriatric patients being affected (10–13). The fact that more cases are being reported in recent years is likely due to increased awareness and easier access to serological testing (10–12). The latency to diagnosis in our case was approximately 4 years, which is to be expected for such mild disease manifestations. Hopefully, the time between disease manifestation and diagnosis can be shortened as more cases are presented.

The gingival lesions as in our case could be misinterpreted as periodontitis. Additionally, our patient had suffered with congenital hypospadias with complicating development of urethral fistula after surgical correction 18 years ago. It remains unclear whether the latter may have already been part of the autoimmune bullous disease activity or actually the trauma initiated the development of autoantigens.

According to literature NC16A BP180 is the most frequent autoantigen in MMP patients followed by reactivity against the LAD-1 ectodomain and laminin 332 (14). Reactivity against BP230 as in our patients is only found in a minority of patients (14–16), while their pathogenetical relevance is disputed, since BP230 is an intracellular antigen (17). Nonetheless, we here present a case with genital MMP involvement due to primarily BP230 autoantibodies, with no similar cases described so far. Serological tests are frequently negative at initial MMP disease stages, thus repeated sampling is required when clinical suspicion for MMP exists (18).

Mucous membrane pemphigoid (MMP) is feared for its treatment refractory course. Our patient responded well to the combination of low dose prednisolone with dapsone, but relapsed mildly when prednisolone was tapered. Instead of dapsone, classical immunosuppressants as mycophenolate or azathioprin can be employed, while rituximab is considered an off-label ultima ratio, especially in severely scarring courses (19–21). Finally, other approaches with intravenous immunoglobulins or omalizumab have been reported to be useful as well (22, 23).

## Data availability statement

The raw data supporting the conclusions of this article will be made available by the authors, without undue reservation.

## Ethics statement

The studies involving human participants were reviewed and approved by Human Ethics Committee University of Freiburg (reference no. 235/15). Written informed consent was obtained from the individual(s) for the publication of any potentially identifiable images or data included in this article.

## Author contributions

FS, FC, and DK had full access to all of the data in the study. FS and DK took responsibility for the integrity of the data and the accuracy of data analysis. All authors read, revised, and approved the manuscript.
